# The Expression of TLR-9, CD86, and CD95 Phenotypes in Circulating B Cells of Patients with Chronic Viral Hepatitis B or C before and after Antiviral Therapy

**DOI:** 10.1155/2015/762709

**Published:** 2015-03-29

**Authors:** Ping-wei Zhao, Liang Ma, Hui-fan Ji, Lei Yu, Jun-yan Feng, Juan Wang, Ming-yuan Liu, Yan-fang Jiang

**Affiliations:** ^1^Key Laboratory of Zoonosis Research, Ministry of Education, The First Hospital, Jilin University, Changchun 130021, China; ^2^Department of Gastroenterology, The First People's Hospital of Changzhou, Third Affiliated Hospital of Suzhou University, Changzhou, Jiangsu 213003, China; ^3^Department of Hepatology Disease, The First Hospital, Jilin University, Changchun 130021, China; ^4^Department of Infectious Disease, The Fourth Hospital of Harbin Medical University, Harbin 150001, China; ^5^Jiangsu Co-Innovation Center for Prevention and Control of Important Animal Infectious Diseases and Zoonoses, Yangzhou 225009, China

## Abstract

*Aims*. This study aimed to assess the differential expression of specific B cell subtypes in patients with chronic viral hepatitis. *Methods*. The frequencies of differential expression of specific B cell subtypes in patients with chronic viral hepatitis and healthy controls were assessed by flow cytometry using monoclonal antibodies specific for CD38, CD27, CD86, CD95, TLR-9, and IgD. The effect of adefovir treatment on B cell subsets in HBV patients was determined. The values of clinical parameters in the patients were also measured. *Results*. The frequency of CD86+ B cells was not significantly different in chronic HBV patients but was higher in HCV patients compared with that in healthy controls. CD95 and IgD levels were lower in HBV and HCV patients than in healthy controls. A significant negative correlation occurred between the proportion of CD95+ B cells and HBV DNA viral load. The frequency of TLR-9 on the B cells in HBV and HCV patients was higher compared with that of healthy controls. After treatment with adefovir, the frequency of CD95 and IgD expressed on B cells was increased in HBV patients. *Conclusions*. Activated B cells and exhausted B cells homeostasis were commonly disturbed in HBV and HCV patients.

## 1. Introduction

Chronic hepatitis infection is a global health concern and an economic burden affecting approximately 500 million people worldwide. Many patients infected with HBV or HCV are at risk for developing chronic liver disease, cirrhosis, and hepatocellular carcinoma [1]. During HBV infection, the interaction between the replicating noncytopathic virus and dysregulatory humoral immunity determines the outcome. Patients with HCV infection display great variability in disease activity and progression. Previous studies have indicated that dynamic interactions between the viruses, hepatocytes, and the host immune systems may determine viral persistence and disease progression, which are displayed in distinct phases [2]. However, the role of humoral immune responses in disease activity and the progression of chronic hepatitis are not well understood.

It is generally believed that T cell immune responses are crucial for viral clearance in HBV/HCV-infected individuals. Both CD4+ and CD8+ T cells are responsible for control of HBV/HCV infection. B cell immunological disturbances play a key role in the development of autoimmunity and cancer and in the success of organ transplantation [1, 3, 4]. However, the role of B cells in HBV and HCV infection is not clear. An in-depth analysis of the B cell phenotype and immunoglobulin production in chronic HCV and chronic HBV infections could help to identify which B cell subpopulations may be enriched in some chronic viral infections [5–10]. This may have implications regarding the ability of subpopulations to differentiate into virus-specific and virus-nonspecific plasma cells.

Some types of B cells, such as antigen-presenting cells (APC), efficiently present antigens to T cells, which results in T cell activation. Moreover, other B cells are also responsible for the production of autoantibodies [11–14]. The expression of CD27 has been particularly useful in distinguishing between memory B cells and naive B cells [15, 16]. However, because CD27 may not be useful for discerning B cell subpopulations reliably in viral hepatitis patients, we evaluated a panel of activation (CD86) and exhaustion (CD95) markers in viral hepatitis patients and normal healthy subjects, using flow cytometry in an attempt to identify and characterize abnormalities in peripheral B cell subset dynamics more completely.

CD38 is a marker of plasma cell maturation [17]. CD86 is a marker of B cell activation. CD95 is mainly present on human activated T-lymphocytes, B-lymphocytes, and malignant lymphoid cells [18]. In humans, TLR-9 is expressed by numerous cells of the immune system such as dendritic cells and B-lymphocytes and leads to activation of the cells initiating proinflammatory reactions that result in the production of cytokines [19, 20]. The purpose of the current study was to measure B cell phenotypic responses during chronic infection and to identify any differences in these responses that might be specifically associated with chronic infections by HBV and HCV, before and after antiviral treatment.

## 2. Materials and Methods

### 2.1. Patients

A total of 35 patients with chronic HBV infection, 50 patients with chronic HCV infection, and 12 spontaneously resolved HCV patients were recruited from the inpatient service and another 17 healthy subjects from the outpatient service of the First Hospital of Jilin University from March 2011 to May 2012. Individual subjects with HBV infection were confirmed positive for HBsAg and detectable HBV virions for at least 12 months  [21]. All hepatitis B patients had the genotype C virus. All HCV-infected patients tested positive for anti-HCV antibody and serum HCV RNA for at least 6 months [22]. Genotyping of hepatitis C patients showed that 26 had genotype 2a, 19 had genotype 1b, and 5 had an unclassified genotype. Spontaneously resolved HCV patients were defined as those patients who lacked HCV RNA at 12 weeks after enrollment without treatment, with prior HCV RNA levels as proof of prior HCV infection [23]. Subjects with positive hepatitis D or HIV infection, or with autoimmune hepatitis or metabolic liver disease, who had received immunosuppressive therapy or antiviral therapy within the past 12 months before entry, were excluded. All patients denied drug use or exposure to hepatotoxins. Their demographic and clinical characteristics of these subjects are shown in Table 1.

HBV patients were treated orally with 10 mg of adefovir dipivoxil (Gilead Science, Foster City, USA) daily for 24 weeks. Their serum alanine aminotransferase (ALT), aspartate aminotransferase (AST), HBsAg, HBsAb, HBeAg, and HBeAb concentrations and serum HBV DNA loads were analyzed (Table 2). The HBV patients all responded to drugs with at least 100-fold reduced serum HBV viral loads after 12 weeks of adefovir dipivoxil treatment. No hepatitis B patients had any adverse reactions following discontinuation of treatment for 24 weeks. HCV patients were treated by subcutaneous injection with 500 million units of a short-acting interferon *α* (SINOGEN, Jinan, China) once every other day for 2 weeks. Individuals with at least a 100-fold reduced serum HCV RNA viral load after 2 weeks of interferon *α* treatment were defined as drug-responsive patients. Those who had less than a 100-fold decline were defined as drug nonresponsive patients (Table 3). The study conformed to the guidelines of the Declaration of Helsinki and was approved by the Human Ethics Committee of Jilin University, Changchun, China. Written informed consent was obtained from each participant, prior to enrollment.

Peripheral blood samples were obtained from individual subjects, and the levels of serums AST and ALT were detected by a Biochemistry Automatic Analyzer (Roche Diagnostics, Branchburg, USA). HCV antibodies were detected by ELISA II (Abbott Laboratories, North Chicago, USA). The levels of serums HBV DNA and HCV RNA loads were measured by a quantitative PCR assay using a luciferase quantitation detection kit with a detection limit of 300 copies/mL (Roche Amplicor, Basel, Switzerland) according to the manufacturer's instructions. The levels of HBV markers, HBsAg, HBsAb, HBeAg, and HBeAb, were determined by a chemiluminescent microparticle immunoassay (CMIA) using an Abbott I 2000 automated chemiluminescence immunoassay analyzer (Abbott Laboratories, Abbott Park, IL, USA). The concentrations of serum HBeAb in individual samples were determined semiquantitatively by a competitive inhibition method, according to the manufacturer's instructions and a previous report [24]. The data are expressed as median (range) of signal OD to cut-off (S/CO). Accordingly, the higher the concentrations of serum HBeAb, the lower the values of S/CO.

### 2.2. PBMC Stimulation with CpGB Oligodeoxynucleotide

Peripheral blood mononuclear cells (PBMCs) were isolated by density-gradient centrifugation using Ficoll-Paque Plus (Amersham Biosciences, Little Chalfont, UK). PBMCs were washed in phosphate-buffered saline (PBS) and diluted at 4 × 10^6^/mL in complete media. RPMI-1640 was supplemented with 10% FCS (FCS, Hyclone, USA) and dispensed (2 × 10^6^/well) in U-bottom 24-well tissue culture plates (Costar, Corning corporation NY, USA). Wells were stimulated with 3 *μ*g/mL CpGB (CpGB oligonucleotide B, R&D Systems, Minneapolis, MN, USA) ±10 ng/mL recombinant IL-2 (R&D Systems, USA). PBMCs were incubated in 5% CO_2_ incubator at 37°C for 3 days [25].

### 2.3. Flow Cytometry

HBV peripheral blood samples were taken at baseline and after adefovir dipivoxil treatment at 12 weeks and 24 weeks; HCV peripheral blood samples were taken at baseline. Human PBMCs at 5 × 10^5^/tube were stained with 10 *μ*L PerCP-anti-CD19, PE-anti-CD38, APC-anti-CD86, PerCP-anti-CD19, PE-anti-CD27, and APC-anti-CD95 (BD Pharmingen, San Diego, USA) at 4°C for 30 min. After fixation and permeabilization, intracellular cytokine staining was performed using FITC-anti-IgD, PE-anti-TLR-9 isotype antibody (BD Pharmingen, San Diego, USA). After washing with PBS, the cells were subjected to flow cytometry analysis using a FACS Caliber (Becton Dickinson) and FlowJo software (v5.7.2). The cells were gated on the forward scatter of living cells and then centered on CD19+ B cells. Subsequently, the CD19+CD86+, CD19+CD38+CD86+, CD19+CD38−CD86+, CD19+CD27+, CD19+CD95+, CD19+CD27+CD95+, CD19+CD27−CD95+, CD19+IgD+, and CD19+TLR-9+ B cells were determined by flow cytometric analysis, and at least 30,000 events per sample were analyzed [25].

### 2.4. Statistical Analysis

Data are expressed as median and range unless specified. The differences between two groups were analyzed by the Wilcoxon rank-sum test and chi-square test using SPSS 14.0 software. The relationship between two variables was evaluated using the Spearman rank-correlation test. A two-side *P* value < 0.05 was considered statistically significant.

## 3. Results

### 3.1. High Prevalence of Activated B Cells and Low Prevalence of Exhausted B Cells in Chronic Viral Hepatitis

**Figure 1:**
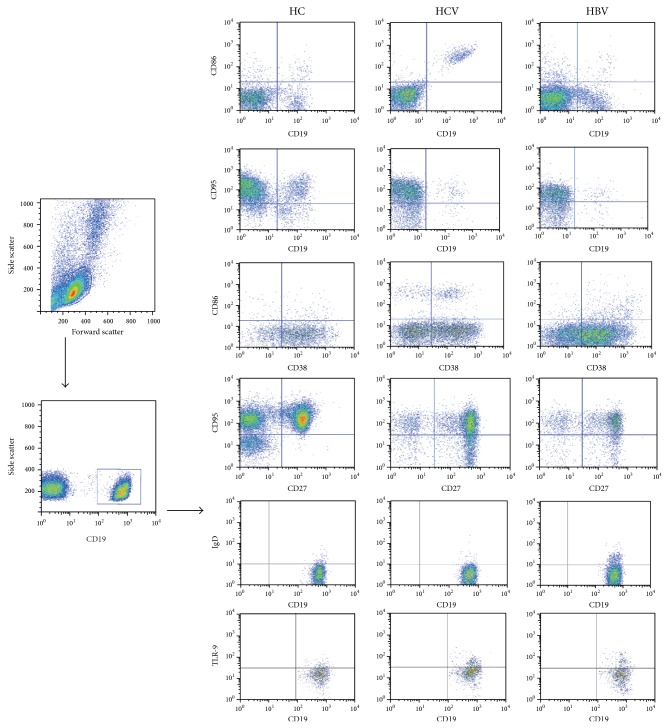
FACS analyses of B cells. Peripheral mononuclear cells were stained with anti-CD19, anti-CD38/27, anti-CD86/95, anti-IgD, anti-TLR-9, or isotype-matched IgG. The cells were gated initially on living lymphocytes (top left) and then on CD19+ B cells (lower left). Subsequently, the frequency of the activation marker (CD86) on total (CD19+), plasma (CD19+CD38+), and nonplasma (CD19+CD38−) B cells, the expression of the exhaustion marker (CD95) on total (CD19+), memory (CD19+CD27+), and naive (CD19+CD27−) B cells, and IgD as well as TLR-9 expressed on total (CD19+) B cells were analyzed by flow cytometry. At least 30,000 events were analyzed for each sample. Data are representatives of different groups of samples from at least two independent experiments.

**Figure 2:**
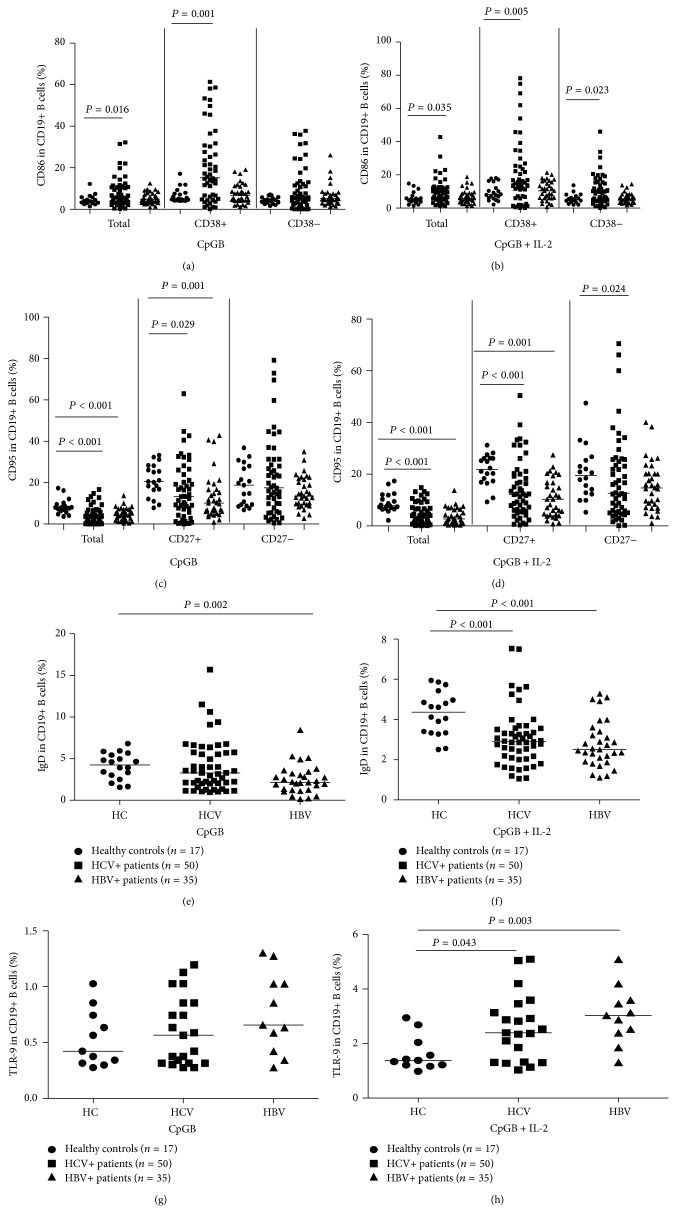
Patients with chronic HBV and HCV infections, and percentages of activated B cells and low percentages of exhausted B cells. B-lymphocytes from HCV patients (HCV, *n* = 50), HBV patients (HBV, *n* = 35), and healthy controls (HC, *n* = 17) were analyzed by flow cytometry* ex vivo* for the expression of the activation marker (CD86), the exhaustion marker (CD95), and IgD. Data is shown as the frequency of the activation marker (CD86) on total (CD19+), plasma (CD19+CD38+), and nonplasma (CD19+CD38−) B cells, the expression of the exhaustion marker (CD95) on total (CD19+), memory (CD19+CD27+), and naive (CD19+CD27−) B cells, and the frequency of IgD and TLR-9 on total (CD19+) B cells stimulated with CpGB ± IL-2. Data are expressed as mean % of individual samples from at least two separate experiments. Data were analyzed by the Wilcoxon rank-sum test. The horizontal lines show the median values.

To evaluate B cell immunity, 35 HBV patients, 50 HCV patients, and 17 healthy subjects were recruited. As shown in Table 1, there were no significant differences in the distribution of age and gender in this population. As expected, the levels of serum ALT, serum AST, and the viral load in HBV and HCV patients were significantly higher than in healthy subjects. Table 1 also shows a temporal window when antibodies against e and s antigen begin to appear, but low levels of antigen e and antigen s remain due to the fact that they have not been completely neutralized. To investigate the potential role of peripheral B cells in HBV and HCV patients, the pretreatment frequencies of peripheral blood CD19+CD86+, CD19+CD38+CD86+, CD19+CD38−CD86+, CD19+CD95+, CD19+CD27+CD95+, CD19+CD27−CD95+, CD19+IgD+, and CD19+TLR-9+ B cells were analyzed by flow cytometry (Figure 1). The proportion of memory B cells was significantly higher in patients with chronic HBV infection (median: 31.09; *P* < 0.006) and significantly lower in patients with chronic HCV infection (median: 16.44; *P* = 0.002) compared with healthy controls (median: 21.52). In HCV patients, a statistically significant negative correlation was found between the proportion of memory B cells and serums ALT (*r* = −0.634, *P* = 0.001) and HCV RNA (*r* = −0.537, *P* = 0.004) but not with serum AST (data not shown). We next evaluated the expression of the activation marker CD86 on total, plasma, and nonplasma B cells and the expression of the exhaustion marker CD95 on total, memory, and naive B cells. The data are summarized in Figure 2. The activation marker CD86 was expressed in a comparable proportion of patients with chronic HBV infection and healthy controls stimulated with CpGB ± IL-2. In HCV patients, CD86 was present at higher levels on total (median: 5.72 versus 3.85, *P* = 0.016) and plasma B cells (15.25 versus 5.34, *P* = 0.001) stimulated only with CpGB (Figure 2(a)). However, after stimulation with CpGB + IL-2, the expressions of CD86 on total (median: 9.31 versus 5.19, *P* = 0.035), plasma (14.85 versus 8.15, *P* = 0.001, *P* = 0.005), and nonplasma B cells (9.31 versus 5.05, *P* = 0.023) in HCV were all higher than those in healthy controls (Figure 2(b)). In HBV infection, the exhaustion marker CD95 stimulated with CpGB ± IL-2 was present at lower levels on total (median: 4.49 versus 7.99, *P* < 0.001; 2.67 versus 7.81, *P* < 0.001, resp.) and memory B cells (median: 10.12 versus 20.54, *P* < 0.001; 10.31 versus 21.75, *P* < 0.001, resp.) than for those in healthy controls (Figures 2(c) and 2(d)). A statistically significant negative correlation was found between the proportion of CD95+ B cells and HBV DNA viral load (*r* = −0.627, *P* = 0.004) but not with serums AST and ALT (data not shown). In HCV infections, CD95 was present at lower levels on total (median: 2.54 versus 7.99, *P* < 0.001) and memory B cells (median: 3.36 versus 7.81, *P* = 0.029) stimulated only with CpGB. However, after stimulation with CpGB + IL-2, the levels of CD95 on total (median: 13.25 versus 20.54, *P* < 0.001), memory (median: 12.12 versus 21.75, *P* < 0.001), and naive B cells (median: 12.67 versus 19.53, *P* = 0.029) in HCV patients were all lower than those in healthy controls. In HBV patients, the expression of IgD on total B cells (median: 2.16 versus 4.27, *P* = 0.002; 2.51 versus 4.37, *P* < 0.001) stimulated with CpGB ± IL-2 was all lower than that in healthy controls (Figures 2(e) and 2(f)). In contrast, in HCV patients, no significant differences in expression of IgD were found between HCV patients and healthy controls stimulated only with CpGB. However, after stimulation with CpGB + IL-2, the level of IgD on total B cells (median: 2.89 versus 4.36, *P* < 0.001) in HCV patients was lower than those in healthy controls. Moreover, in both HBV (median: 3.26 versus 1.38, *P* = 0.003) and HCV (median: 2.39 versus 1.38, *P* = 0.043) patients, the functional marker TLR-9 stimulated with CpGB + IL-2 was present at higher levels on total B cells than for those in healthy controls (Figures 2(g) and 2(h)).

### 3.2. High Prevalence of Activated B Cells in Interferon-Responsive HCV Patients

**Figure 3:**
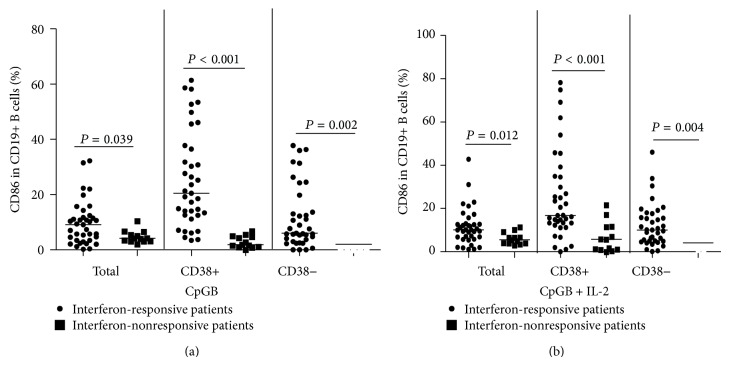
The expression of activation marker (CD86) in interferon responsive and nonresponsive HCV patients at baseline. A total of 50 HCV patients were treated with interferon for 2 weeks, and the frequency of CD86+ B cells in peripheral blood was determined by flow cytometry analysis at baseline: (a) the expression of the activation marker (CD86) on total (CD19+), plasma (CD19+CD38+), and nonplasma (CD19+CD38−) B cells stimulated with CpGB and (b) the expression of the activation marker (CD95) on total (CD19+), plasma (CD19+CD38+), and nonplasma (CD19+CD38−) B cells stimulated with CpGB + IL-2. Data were analyzed by the Wilcoxon rank-sum test. The horizontal lines indicate the median values of different groups. Data are expressed as mean % of individual samples from at least two separate experiments.

We analyzed the frequency of B cells in interferon-responsive and interferon-nonresponsive HCV patients at baseline (Table 2). The expression of the activation marker CD86 in interferon-responsive patients was significantly higher than that in interferon nonresponsive patients at baseline (CpGB median: CD19+CD86+ B cell 9.07 versus 4.22, CD19+CD38+CD86+ B cell *P* = 0.039; CD19+CD38−CD86+ B cell 1.91 versus 20.48, *P* < 0.001; 2.05 versus 6.05, *P* = 0.002Figure 3(a); CpGB+IL-2 median: CD19+CD86+ B cell 10.1 versus 5.52, CD19+CD38+CD86+ B cell *P* = 0.012; 16.80 versus 5.81, *P* < 0.001; CD19+CD38−CD86+ B cell 10.01 versus 4.13, *P* = 0.004Figure 3(b)). Twelve HCV-infected patients were positive for HCV antibody but negative for serum HCV RNA, at 12 weeks after enrollment without treatment. We defined these individuals as spontaneously resolved HCV patients. We found that there were no significant differences in expression of CD86 and CD95 on B cells between spontaneously resolved HCV patients and chronic HCV patients. Considering HCV genotypes (2a = 26, 1b = 19, unclassified = 5), we found there was no significant difference in the frequency of peripheral B cells among different HCV genotypes (data not shown).

### 3.3. Serological Markers of HBV Infection Altered by Adefovir Dipivoxil Treatment

HBsAg, HBsAb, HBeAg, and HBeAb levels were examined in patients' serum samples at baseline and after 12 and 24 weeks of adefovir dipivoxil treatment (Table 3). Basal serums HBsAg and HBeAg levels were high in all the patients but decreased rapidly following the adefovir dipivoxil treatment. Serum HBsAb levels showed a tendency to increase after 12 and 24 weeks of adefovir dipivoxil treatment. HBeAb concentrations of each pooled serum were determined by a semiquantitatively competitive inhibition method. In this assay, the higher the HBeAb concentration, the lower the value of S/CO. The results showed that the HBeAb concentration of each pooled serum was significantly increased after 12 and 24 weeks of adefovir dipivoxil treatment compared to baseline.

### 3.4. Treatment with Adefovir Dipivoxil Significantly Changed Frequency of B Cell Subsets in HBV Patients

**Figure 4:**
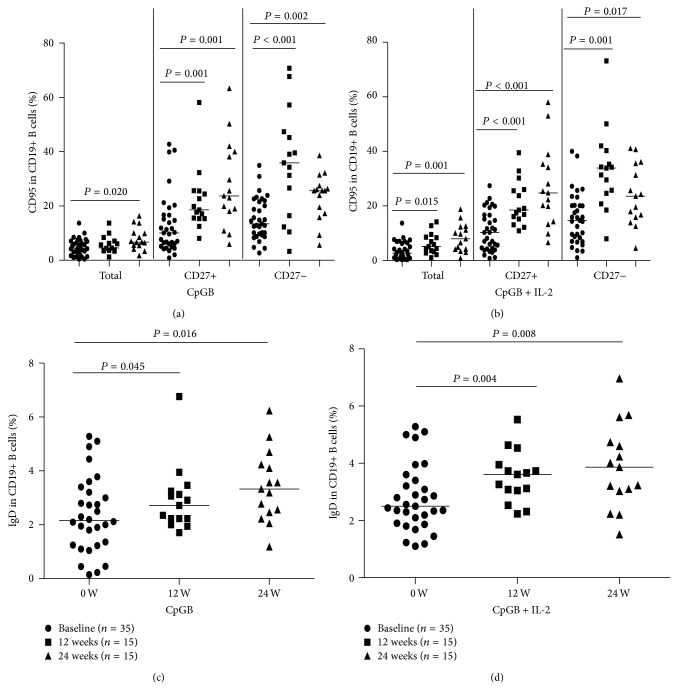
Treatment with adefovir dipivoxil modulates the frequency of CD95+ B cells in HBV patients. A total of 15 HBV patients were treated with adefovir dipivoxil for 24 weeks, and the frequency of CD95+ B cells and IgD+ B cells in peripheral blood was determined by flow cytometry analysis at baseline and after treatment at 12 and 24 weeks: (a) the expression of the exhaustion marker (CD95) on total (CD19+), memory (CD19+CD27+), and naive (CD19+CD27−) B cells stimulated with CpGB, (b) the expression of the exhaustion marker (CD95) on total (CD19+), memory (CD19+CD27+), and naive (CD19+CD27−) B cells stimulated with CpGB+IL-2, (c) the frequency of IgD on total (CD19+) B cells stimulated with CpGB, and (d) the frequency of IgD on total (CD19+) B cells stimulated with CpGB+IL-2. Data were analyzed by the Wilcoxon rank-sum test. The horizontal lines indicate the median values of different groups. Data are expressed as mean % of individual samples from at least two separate experiments.

Fifteen HBV patients were treated with adefovir dipivoxil for 24 weeks, and their percentages of B cells subsets were characterized before and after drug treatment. Following treatment with adefovir dipivoxil, all patients had reduced levels of serum ALT, serum AST, and HBV DNA loads compared with those levels before treatment (Table 3). Compared with the analysis of B cells before treatment, the exhaustion marker CD95 on memory (median: 18.55 versus 10.12, *P* = 0.001) and naive (median: 35.89 versus 13.44, *P* < 0.001) B cells stimulated with CpGB alone were increased after treatment with adefovir dipivoxil for 12 weeks (Figure 4(a)). After stimulation with CpGB + IL-2, the levels of CD95 on total (median: 5.08 versus 2.67, *P* = 0.015), memory (median: 18.51 versus 10.31, *P* < 0.001), and naive (median: 33.93 versus 14.67, *P* = 0.001) B cells were increased (Figure 4(b)). However, in comparison with adefovir dipivoxil treatment for 12 weeks, after treatment with adefovir dipivoxil for 24 weeks, there was no significant difference in CD95 levels in chronic HBV patients (Figures 4(a) and 4(b)). In comparison with baseline, the expression of IgD on total B cells (median: 2.72 versus 2.16, *P* = 0.045, 3.33 versus 2.16, *P* = 0.016; 3.61 versus 2.51, *P* = 0.004, 3.86 versus 2.51, *P* = 0.008, resp.) stimulated with CpGB ± IL-2 was significantly increased after treatment with adefovir dipivoxil for 12 and 24 weeks (Figures 4(c) and 4(d)). Clearly, treatment with adefovir dipivoxil decreased the replication of HBV DNA and restored the frequency of CD95+ B cells and IgD+ B cells in HBV patients.

## 4. Discussion

The current study revealed disturbed homeostasis of peripheral B cell subsets during chronic infection with HBV as well as with HCV. The frequency of peripheral blood CD86+ B cells in HCV patients was significantly higher than that of healthy controls which is in agreement with the data of Oliviero et al. [26], with some notable differences. In contrast, the exhaustion marker (CD95) and IgD were present at lower levels in chronic viral hepatitis patients than in healthy controls. More importantly, after treatment with adefovir dipivoxil, the frequencies of B cells exhaustion marker (CD95) and IgD on total B cells in HBV patients were significantly increased. These findings indicated that B cells might participate in the HBV-related immune responses, which provide several important insights into the pathobiology of B cells in newly diagnosed HBV or HCV patients. Moreover, our findings may provide new insights that may aid in the design of new immunotherapies for treatment of HBV or HCV in the clinic.

In contrast to the report of Oliviero et al. [26], the current data on patients with HBV infection showed a higher proportion of memory B cells in profiles in contrast to that of HCV-infected patients. These results show that HBV stimulation induces a strong increase in memory B cells. In HCV patients, a statistically significant negative correlation was found between the proportion of memory B cells and serums ALT and HCV RNA, suggesting that HCV infection induces a strong depletion in memory B cells. Oliviero et al. [26] also tested B cells from patients with chronic hepatitis and showed a significantly increased expression of activation molecules (CD69, CD71, and CXCR3), but not CD86. In accordance with this report, we found that CD86 was expressed in a comparable proportion of patients with chronic HBV infection and healthy controls. In addition, we showed that exhausted B cells were, in most cases, equally distributed between memory and naive cells. The exhaustion marker CD95 was present at lower levels in chronic viral hepatitis than in healthy controls. A statistically significant negative correlation was found between the proportion of CD95+ B cells and HBV DNA viral load. Most of these defects have been considered hallmarks of the chronic phase of viral hepatitis infection. CD95 expression on circulating B cells has previously been described to be increased in patients with active systemic lupus erythematosus (SLE) [27], indicating that increased Fas expression results in a higher susceptibility for Fas-mediated apoptosis, which might contribute to the increased levels of apoptotic lymphocytes in SLE patients. During persistent virus infections, continuous B cell activation may result in an accumulation of exhausted B cells that have defective function and increased expression of inhibitory receptors as described for HIV [28, 29]. However, the B cell exhaustion marker (CD95) in our patients was present at lower levels than in healthy controls, arguing against the hypothesis that B cells are severely impaired in most chronic viral infections. This would explain why patients with chronic HBV and HCV infections maintain the ability to produce antibodies to recall antigens and are able to respond to soluble-protein vaccines. In HBV patients, the expression of IgD was lower than in HCV patients and healthy controls. IgD is a marker of B cell differentiation, development, and maturation. The expression of IgD has been shown to gradually disappear from the surfaces of activated B cells and memory B cells [30]. This is consistent with the increases in activated B cells and memory B cells found in HBV patients in the current study. The B cell functional marker, TLR-9, was found to be present at higher levels in HBV and HCV patients compared to healthy controls, indicating a possibility that TLR-9 signals play a protective role during hepatitis virus infection. TLR-9 expression is upregulated after HBV infection, which may result in stimulation of B cells to switch isotype to IgG2a against viral infection [19]. However, it is also possible that TLR-9 signals lead to activation of the B cells initiating proinflammatory reactions that result in the production of cytokines such as type-I interferon and IL-12 [20]. Moreover, Dejager and Libert showed that TLR-9 signals caused hepatocyte exhaustion and hepatic failure by promoting TNF-*α* production [31]. It is worth noting that there were no significant differences in B cell subsets between spontaneously resolved HCV patients and chronic HCV patients. This further illustrates that the expression of B cells was not associated with HCV RNA load. The expression of the activation marker CD86 in interferon-responsive patients was significantly higher than that in interferon nonresponsive patients at baseline, suggesting that CD86 may be an important factor in determining whether HCV patients are interferon-responsive. Moreover, interferon might increase the expression of CD86 in interferon-responsive patients by other mechanisms. Taken together, these various types of B cells may be useful as markers for the intervention of HBV or HCV in the clinic.

Adefovir dipivoxil is a potent antiviral agent, and treatment with this agent can effectively inhibit the replication of HBV in the majority of chronic hepatitis B (CHB) patients. Our previous studies have shown that treatment with adefovir dipivoxil enhanced TFH cell immunity which was associated with the inhibition of HBV replication in CHB patients [25, 32, 33]. Fazilleau et al. found that TFH cells were involved in activation of B cells to become professional antibody producers [34]. In the current study, we further examined the impact of treatment with adefovir dipivoxil on the frequency of CD95+ B cells and IgD+ B cells in patients with chronic viral hepatitis and found that treatment with adefovir dipivoxil for 12 weeks not only significantly decreased the concentrations of serum ALT, serum AST, HBV virus, HBsAg, and HBeAg, but also dramatically increased the concentrations of HBsAb and HBeAb and the frequency of CD95+ B cells and IgD+ B cells in the drug-responding HBV patients. This suggested that B cells play an important role in the process of immune response. Of note, a statistically significant negative correlation was found between the proportion of CD95+ B cells and HBV DNA viral load. The increased frequency of CD95+ B cells may be due to dramatically reduced HBV DNA virus loads, possibly due to preferentially deleterious effects of HBV on memory B cells.

In summary, our data suggest that B cell homeostasis is disturbed in patients with chronic HBV and HCV infections. We recognized that our study had limitations, including a small sample size and the lack of convincing markers and functional studies of different subsets of lymph node B cells. Thus, a more comprehensive analysis of the properties of B cell subsets at various infection stages is needed to provide further insights into B cell responses and dysregulation in chronic viral hepatitis.

## Figures and Tables

**Table 1:** Demographic characteristics and clinical features of participants.

Parameters	HBV	HCV	Healthy controls
Number	35	50	17
Age (years)			
Mean ± SD	45 ± 10	43 ± 10	44 ± 9
Median (range)	40 (19–62)	45 (27–69)	49 (42–54)
Sex *N* (%)			
Male	30 (85,8)	29 (48)	9 (52,9)
Female	5 (14,2)	21 (42)	8 (47,1)
Viremia (log_10_ copies/mL)			
Median (range)	8,4 (5,7–9,8)^*^	5,9 (1,2–7,5)^*^	NA
ALT (U/L)			
Median (range)	219 (12–914)^*^	44,1 (11,2–274,90)^*^	16 (4–29)
AST (U/L)			
Median (range)	117 (22–221)^*^	44,6 (13,9–262)^*^	16 (8–32)
HBsAg (IU/mL)			
Median (range)	4697,68 (1243,65–55925,04)^*^	/	NA
HBsAb (mIU/mL)			
Median (range)	0,06 (0–3,71)^*^		NA
HBeAg (S/CO)			
Median (range)	3233,3 (3,88–4094,10)^*^	/	NA
HBeAb (S/CO)			
Median (range)	16.74 (0,02–46,74)^*^		NA

Normal values: ALT ≤ 40 IU/L; AST ≤ 40 IU/L; HBV DNA ≤ 3 log_10_copies/mL; HCV RNA ≤ 3 log_10_copies/mL; ^∗^ *P* < 0.05 HBV/HCV versus healthy controls.

**Table 2:** Effects of interferon treatment on clinical profiles of HCV patients.

Group	Drug-responsive (*n* = 37)		Drug-nonresponsive (*n* = 13)	
	Before	After	Before	After
ALT (U/L)				
Median (range)	56,5 (13,2–229,9)	29,6 (13–69)^*^	53,2 (18,4–144,2)	36,3 (15–127)^*^
AST (U/L)				
Median (range)	34,2 (15,4–111,3)	20,5 (13–47)^*^	35,3 (27,1–80)	25,2 (16–65)^*^
HCV RNA (log_10_ copies/mL)				
Median (range)	5,4 (5,1–6,9)	2,7 (0,3–3,1)^*^	5,5 (5,2–6,7)	5,1 (4,8–6,7)

Data are expressed as median (range) or real case numbers.

^∗^ *P* < 0.05 versus before treatment.

**Table 3:** Treatment with adefovir dipivoxil modulates clinical profiles of HBV patients.

Parameters	0 weeks	12 weeks	24 weeks
HBV DNA (log_10_ copies/mL)			
Median (range)	8,4 (5,7–9,8)	3,5 (1,9–5,8)^*^	2,5 (1,8–4,1)^*^
ALT (U/L)			
Median (range)	219 (12–914)	45 (16–171)^*^	24 (10–148)^*^
AST (U/L)			
Median (range)	117 (22–221)	27 (18–123)^*^	22 (16–54)^*^
HBsAg (IU/mL)			
Median (range)	4697,68 (1243,65–55925,04)	3478,45 (890,34–17118,09)	1729,56 (356,71–12315,86)^*^
HBsAb (mIU/mL)			
Median (range)	0,06 (0–3,71)	0,4 (0–4,41)	3,56 (0,52–8,56)^*^
HBeAg (S/CO)			
Median (range)	3233,3 (3,88–4094,10)	539,62 (0,47–2345,67)^*^	21,3535 (0,35–766,13)^*^
HBeAb (S/CO)			
Median (range)	16,74 (0,02–46,74)	1,26 (0,01–35,67)^*^	0,46 (0–16,23)^*^

Normal values: ALT ≤ 40 IU/L; AST ≤ 40 IU/L; HBV DNA ≤ 3 log_10_ copies/mL.

^∗^ *P*< 0.05 patients after treatment with adefovir dipivoxil versus patients at baseline.

## Abbreviations

HBV: Chronic hepatitis B
HCV: Chronic hepatitis C
ALT: Alanine aminotransferase
AST: Aspartate aminotransferase
CMIA: Chemiluminescent microparticle immunoassay
S/CO: Signal OD to cut-off
PBMCs: Peripheral blood mononuclear cells
PBS: Phosphate-buffered saline
SLE: Systemic lupus erythematosus
CHB: Chronic hepatitis B.
